# Preparation of Biodegradable Polyethylene Glycol Dimethacrylate Hydrogels via Thiol-ene Chemistry

**DOI:** 10.3390/polym11081339

**Published:** 2019-08-13

**Authors:** Gavin Burke, Zhi Cao, Declan M. Devine, Ian Major

**Affiliations:** Materials Research Institute, Athlone Institute of Technology, Dublin Road, Co. Westmeath N37 HD68, Ireland

**Keywords:** PEGDMA, thiol monomers, thiol-ene, click chemistry, photopolymerisation, biodegradability

## Abstract

Through the control of the molecular weight, water content and monomer concentration, polyethylene glycol dimethacrylate (PEGDMA) based hydrogels have been adapted for numerous applications, including as structural scaffolds, drug delivery vehicles and cell carriers. However, due to the low biodegradability rates, the use of PEGDMA in tissue engineering has been limited. Thiol-based monomers have been shown to improve the degradation rates of several PEG-based hydrogels, though their impact on several material properties has not been as well defined. In this work, several mercaptopropianoates, as well as mercaptoacetates, were mixed with PEGDMA and copolymerized. Following an initial polymerization check, it was determined that mercaptoacetate-based thiol monomers did not polymerize in the presence of PEGDMA, whereas mercaptopropionates were more successful. The wettability, and the compressive and tensile strength, in addition to the thermal properties, were determined for successfully copolymerized samples via a combination of differential scanning calorimetry, dynamic mechanical analysis, unconfined compression, and goniometry. Further study determined that dipentaerythritol hexa(3–mercaptopropionate) (DiPETMP) successfully enhanced the biodegradability of PEGDMA.

## 1. Introduction

Tissue engineering is a multidisciplinary field, wherein the combination of growth factors, scaffolds and cell encapsulation allows for the replication of properties found in a tissue or organ present within the body. The technique can replace damaged tissues or facilitate the regeneration of damaged tissues [1]. In the past, there have been many hydrogels of both synthetic and natural origins employed for tissue engineering scaffolds, and there are advantages and disadvantages to both options. Polyethylene dimethacrylate (PEGDMA) is a synthetic polymer that has seen increasing use over the past 25 years and is advantageous for tissue engineering due to the degree of control that can be exerted over its properties. Through the control of its molecular weight (M_w_), water content and monomer concentration, the properties of PEGDMA can be altered to suit multiple tissue engineering applications ranging from tissue scaffolds to drug delivery vehicles to cell carriers [2,3]. Although PEGDMA has been proven to have highly desirable traits due to the versatility of its properties, it suffers from a significant limitation in its lack of degradability over a biologically relevant timeframe. As degradation of biomaterial scaffolds is a critical factor in successful tissue regeneration, it is imperative for the degradation rate to be a prime consideration. Several issues can occur if the rate of polymer degradation is not correctly controlled; if the degradation rate is too slow, it can impair tissue repair, whereas there is an increased likelihood of inflammation if the degradation rates are too fast [4]. Many different approaches are currently being employed to improve the degradation rates such as copolymerisation with degradable natural polymers and the addition of biodegradable peptide linkages [5]. However, these methods suffer from limitations, including increased immunological responses, as well as poor mechanical performance [6].

**Figure 1:**
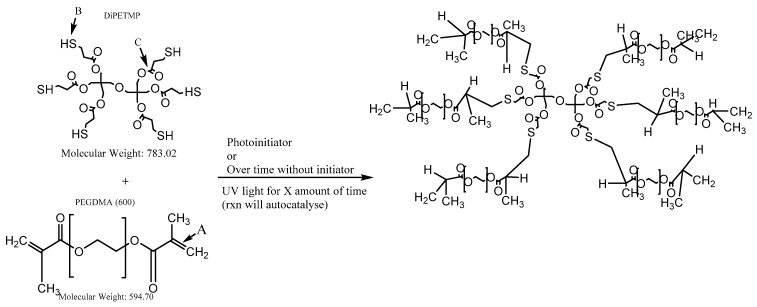
Photopolymerisation of polyethylene glycol dimethacrylate (PEGDMA) and dipentaerythritol hexa(3–mercaptopropionate) (DiPETMP); Arrows A and B respectively point to the reactive points for PEGDMA and DiPETMP; Arrow C points to one of the ester bonds that is broken during the hydrolytic degradation.

One frequent approach to improving the degradability of PEG-based hydrogels has been the utilisation of thiol-ene reactions. Thiol-ene reactions have been well studied in the past [7,8,9] and function on the premise of a thiol (S–H) group readily reacting with a C=C bond to form a thio-ether (as shown in Figure 1, where a PEGDMA-based hydrogel has been incorporated with dipentaerythritol hexa(3–mercaptopropionate) (DiPETMP)). There are several key advantages to the use of thiol-ene reactions, in addition to those typically associated with photopolymerisation. Thiol-ene hydrogels experience a decreased shrinkage, which is commonly associated with PEG photopolymerisation. They have great flexibility in that they can be initiated by radical-mediated additions or by Michael type addition reactions and do not suffer from oxygen inhibition, which is a common limitation of acrylate polymerisations [10,11,12].

Furthermore, thiol-ene reactions fit the criteria of being considered click reactions. Namely, they maintain a very high rate of C=C double bond conversion during polymerisation [13]. Thiol-based monomers have been shown to improve the degradation rates of several PEG-based hydrogels synthesised from PEGDA, PEG-PLA and PEG-Norbornene [12,14,15]. The processes of thiol-ene degradation have been well-defined at this point, operating as a pseudo-first-order degradation process through the hydrolytic degradation of the ester linkage [11]. Although the effects of thiols on the degradation rates of PEG-based hydrogels have been previously studied, there has been less work regarding their impact on the mechanical performance.

For thiols to be employed for the improvement of PEG-based hydrogel degradation rates, it is necessary to understand their effect on PEG-based hydrogels’ mechanical and thermal properties. For tissue engineering applications, it has been shown that differences in the mechanical strength of the extracellular matrix upon which cells are grown and subjected to, can result in mesenchymal stem cells differentiating into completely different cell types, such as neural cells, muscle cells and bone cells [16,17,18]. Furthermore, if hydrogels are to be used for biomedical applications, it becomes important to understand whether there are thermal transitions occurring at physiologically relevant temperatures which could alter the mechanical properties and affect the function of the hydrogel. With this in mind, we set out to polymerise PEGDMA in a 1:1 molar ratio with several thiol-based monomers. Extensive studies have been performed on some thiol monomers, notably pentaerythritol tetra–(3–mercaptopropionate) (PETMP) [19,20,21,22,23]. However, their properties when photopolymerised with and compared to PEGDMA, have not been fully elucidated. In this study, we first set out to determine which of ten different thiol-based monomers could be successfully copolymerised with PEGDMA 600 via UV photopolymerisation. Following this, the properties of the PEGDMA-thiol hydrogels were compared with PEGDMA hydrogels prepared as in previous studies [24], to determine the effect of thiol incorporation on performance. Subsequently, the PEGDMA-DiPETMP hydrogel was subjected to an accelerated degradation study to ascertain to what extent the PEGDMA degradability had been enhanced. 

## 2. Materials and Methods

### 2.1. Materials

The macromolecular monomer polyethyleneglycol dimethacrylate M_W_ 600 was purchased from Polysciences, Hirschberg an der Bergstrasse, Germany. The photoinitiator Irgacure 2959 was supplied by Ciba Specialty Chemicals, Basel, Switzerland. PETMP, DiPETMP, pentaerythritol tetramercaptoacetate (PETMA), ethoxilated-trimethylolpropan tri(3–mercaptopropionate) (ETTMP 700), ethoxilated-trimethylolpropan tri(3–mercaptopropionate) (ETTMP 1300), trimethylol-propane Tri (3–mercaptopropionate) (TMPMP), tris[2–(3–mercapto- propionyloxy) ethyl]isocyanurate (TEMPIC), glycol dimercaptoacetate (GDMA) glycol di(3–mercaptopropionate) (GDMP) and polycaprolactone tetra(3–mercaptopropionate) (PCL4MP 1350), were supplied by Bruno Bock, Marschacht, Germany. A sodium hydroxide (NaOH) solution was prepared from solid NaOH tablets (purchased from Fisher Scientific, Dublin, Ireland). All materials were used as received.

### 2.2. Hydrogel Fabrication

PEGDMA-thiol mixtures were photopolymerized using a UV curing system (Dr. Gröbel UV-Electronik GmbH, Ettlingen, Germany) in an irradiation chamber with a controlled radiation source consisting of 20 UV-tubes in the spectral range of 315–400 nm at an average intensity of 10–13.5 mW/cm^2^. The pre-cured mixtures were prepared by combining the desired amounts of macromolecular monomers and Irgacure 2959 photoinitiator, as shown in Table 1. The batches were placed in 50 mL beakers, and mixed using a magnetic stirrer at 200 rpm for 20 min at room temperature. The solutions were pipetted into silicone moulds, and photopolymerisation was carried out for 10 minutes, after which time the polymerisation had occurred.

### 2.3. Polymerisation Verification

Post-UV exposure samples were viewed as having polymerised if they had formed a hydrogel; the polymerised samples were removed from the polymer moulds, washed to remove excess water and stored. The un-polymerised samples were exposed to UV again to ensure that delayed polymerisation was not occurring, and after 30 min of further exposure any unreacted monomers were considered as having unsuccessfully polymerised.

### 2.4. Chemical Analysis

Attenuated total reflectance Fourier transform infrared spectroscopy (ATR-FTIR) (Perkin Elmer, Waltham, MA, USA) was employed to analyse the presence of unreacted monomers post polymerisation. Prior to the analysis, all the samples were dried in a vacuum oven at 80 °C. After that, the samples were cut and placed under the crystal of the ATR/FTIR spectrometer. All data were recorded at 21 °C, in the spectral range of 4000–650 cm^−1^, utilising a four scan per sample cycle and a fixed universal compression force of 80 N (n = 4).

### 2.5. Compression Testing

Testing was carried out using a screw-driven mechanical testing machine (Lr10K, Lloyd Instruments, Bognor Regis, UK)fitted with a 2.5 kN load cell (n = 4). The samples were equilibrated in phosphate buffer (pH 7.4) at room temperature for 48 h before testing, and an unconfined compression was carried out at a speed of 1 mm/min using samples that were 28 mm in diameter and 2.5 mm in height. A pre-load of 5 N was employed, and the samples were compressed to a 70% strain. Stress-strain curves were generated, from which Young’s modulus was calculated based on the linear section of the graph, with the toe region (if any was present) being ignored. For each hydrogel sample, the stress at limit was calculated based on the load at the compressive limit.

### 2.6. Wettability Measurement

Wettability measurements were carried out using the water contact angle method. Following removal from the phosphate buffer solution (PBS), the samples were rinsed with deionized H_2_O and blotted dry with filter paper. A 10 μL drop of deionized water was placed on the surface of each sample, an image of the droplet was recorded, and from this image a contact angle was calculated. Contact angles were carried out in quintuplicate on each sample, with 3 of each hydrogel being tested for wettability. 

### 2.7. Thermal Properties

To determine if the thermal properties are impacted by thiol addition, the glass transition temperature of both PEGDMA and PEGDMA-thiol hydrogels were analyzed using differential scanning calorimetry (DSC) (TA Instruments, New Castle, Delaware, USA). Between 8 and 12 mg of each sample was weighed using the Sartorious microbalance, heated at a rate of 20 °C/min from room temperature to 110 °C, cooled to –70 °C using the cooling compartment of a DSC machine and reheated to 200 °C at a rate of 5 °C/min. From the subsequent thermographs, it was possible to determine the glass transition temperature for each sample (n = 2). Volatiles were removed from the purging head with nitrogen at a rate of 30 mL/min. The calibration of the instrument was performed using indium as the standard.

### 2.8. Surface Properties

Scanning electron microscopy (SEM) was performed on a Mira SEM (TESCAN, Oxford Instruments, Abingdon, UK) using a range of magnifications. Samples 5 mm in diameter and 1 mm in height were sectioned into two pieces. Thereafter, the surface of the samples and the cross-section were examined. As a first step, the samples were placed on an aluminium stub and were gold-coated using a Bal-tec SCD 005 sputter coater for 110 sec at 0.1 mBar vacuum before testing. The investigation of the dimensions of the PEGDMA samples were performed with the energy dispersive x-ray (EDX) system.

### 2.9. Swelling Studies

Swelling experiments were performed on samples with a diameter of ≈12.5 mm, height of ≈1.15 mm and a beginning weight between 170 and 245 mg (n = 5) in PBS (pH 7.4). After the photopolymerisation, the samples were weighed and placed in McCartney bottles filled with 15 ml PBS until equilibrium swelling was reached and were vacuum dried at 80 °C, as described previously [24]. The percentage swelling of the samples was calculated using the formula:

(1) $$Swelling\;\left(\%\right)=\left(\frac{W_{s}-W_{d}}{W_{d}}\right)\times \frac{100}{1}$$

where *W_s_* and *W_d_* are the weights of the hydrogels in the swollen state and the dried state, respectively (with five replicates or n = 5).

The gel fraction was calculated using the formula outlined by others previously [25], where Wredry refers to the weight after the sample has been swollen and subsequently dried:

(2) $$Gel\;fraction\;=\left(\frac{Wd}{Wredry}\right)\times \frac{100}{1}$$

### 2.10. Dynamic Mechanical Analysis

A dynamic mechanical analysis was performed using a Q-800 analyser (TA Instruments, New Castle, DE, USA) fitted with a Peltier temperature control. The samples were tested within 72 h of preparation at a temperature of 37 °C using a tensile mode tester, where the samples were in the swollen equilibrium state. Prior to testing, all samples were blotted free of water using filter paper in an attempt to minimise slippage. A compression load of 5.0 ± 0.2 N was exerted on the samples during the testing. Storage/elasticity (G′) and loss (G″) moduli were obtained under dynamic conditions. Dynamic strain sweep test experiments were performed at a constant frequency of 1 Hz, with a percentage strain ranging from 1.80 × 10^−4^ to 1.0 × 10^−3^.

### 2.11. Raman Characterisation

Raman spectra were obtained similarly to [26] using a Renishaw inVia Raman confocal microscope (Renishaw Instruments, Gloucestershire, UK), with a 785 nm laser excitation operating at 300 mW. Spectra were collected between 100 and 3200 cm^−1^ over the course of 8 spectral scans (run time of 10 s each). A 20× lens with a 50 μm laser spot size and a path grating of 1200 lines/mm (633/780) were used throughout. Similar to the work carried out by others [27], the differences in peak heights at 1640 cm^−1^ were used as an indicator of the degree of crosslinking, and peaks were normalised to the 1470 cm^−1^ peak representative of CH_2_ bending.

### 2.12. Accelerated Degradation Study

To assess whether the addition of thiols improve the degradation rates of PEGDMA, a comparative accelerated degradation study was carried out. Cylindrical samples of PEGMA and PEGDMA-thiol composites with an average weight of 200 mg were stored in either 5 mM or 5M NaOH, and their change in weight was monitored following the procedures outlined by Browning and Cosgriff-Hernandez et al., 2014 and Lam and Hutmacher et al., 2008, respectively [28,29]. To highlight the degree of breakdown, pictures were taken weekly after the first month of degradation, and the experiment continued until it was no longer possible to retrieve samples from their NaOH solutions.

## 3. Results

### 3.1. Polymerisation Verification

**Figure 2:**
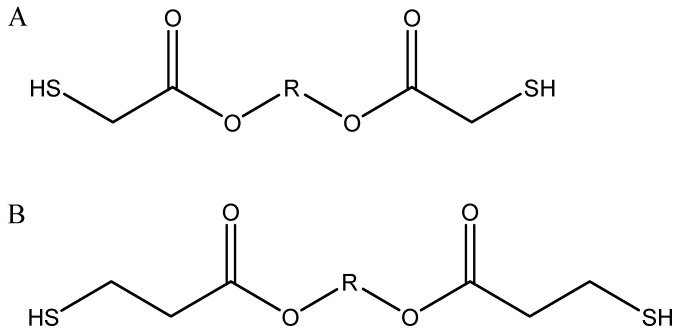
(**A**) Mercaptoacetate structure, and (**B**) Mercaptopropionate structure

Following 10 min of UV exposure, it was found that three thiol-ene combinations produced a hydrogel: PETMP, DiPETMP and GDMP. All other thiols were exposed to UV for an additional 30 min, resulting in no formation of hydrogels. Following removal from the UV chamber, unreacted monomers were stored in the dark for one week to observe whether self-polymerisation would occur. The ETTMP 1300-PEGDMA hydrogels self-polymerised over time and were tested for their material properties. From the results in Table 2, it is difficult to predict whether a successful polymerisation will occur, as both the number of reactive groups present per monomer and the molecular weight of thiols varies greatly in successfully polymerised thiols. PETMP, DiPETMP and GDMP have a similar –SH group content to each other; however, these are also comparable to non-polymerized monomers such as TMPMP, and there are both monomers with a higher and lower –SH content. However, it should be noted that no mercaptoacetates successfully polymerised and no thiol-based macromolecular monomer polymerised by UV exposure (ETTMP 1300 polymerised over time). For comparison, the main difference between GDMA and GDMP can be seen in Figure 2A,B respectively (with the R group representing identical monomer structures), namely the extra carbon chain in the monomer backbone increasing the distance between the thiol and ester groups. 

### 3.2. Chemical Analysis

**Figure 3:**
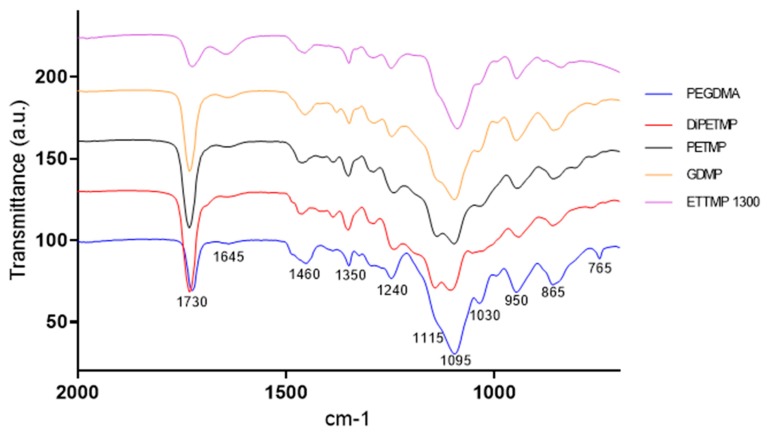
The FTIR spectra for PEGDMA and PEGDMA-thiol samples post polymerization.

The FTIR results can be seen in Figure 3; across most samples there is no presence of the peaks at 815 cm^−1^ or the peak at 1167 cm^−1^, indicative of C–H bending of the H_2_C=CH–C=O group and C–O–C stretching, respectively [31,32,33]; this suggests that complete polymerisation occurred across all hydrogels. In the DiPETMP samples, there was a peak at 1142 cm^−1^; however, this peak can be explained by DiPETMP’s chemical structure containing a C–O–C bond at its centre (see Figure 1). With the GDMP and ETTMP 1300 samples, pronounced peaks were found at 1637 cm^−^^1^ (peak representative of the C=C bending of the CH_2_=CH groups [34]), indicating that a large amount of PEGDMA monomer did not react with the thiol monomer. When considering that ETTMP 1300 polymerised over time instead of through UV exposure, there is an increased likelihood of ETTMP 1300 self-polymerising with large amounts of PEGDMA remaining unreacted. No peak in the 2550–2600 cm^−1^ range indicates the disappearance of all S–H bonds, further evidence that complete thiol convergence had occurred [35], whereas the lack of peaks falling in the 710–685 cm^−1^ range associated with the formation of the C–S–C bond during polymerisation may be too weak to be detected by IR methods [35,36].

### 3.3. Compression Testing

**Figure 4:**
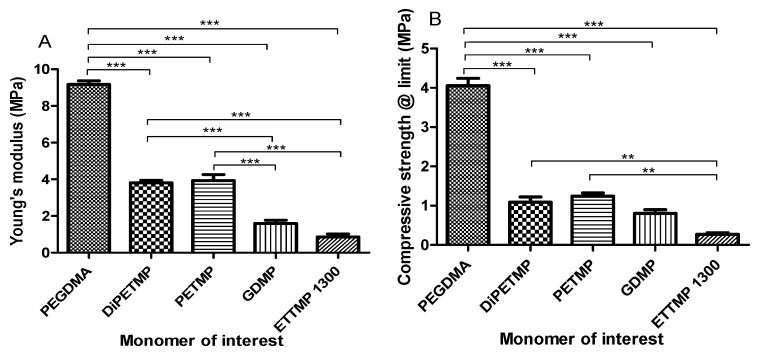
The compressive properties of PEGDMA-thiol hydrogels showing (**A**) the compressive modulus at limit (MPa) and (**B**) Young’s modulus (MPa).

In Figure 4, the compressive strength at limit and Young’s modulus are shown for each hydrogel. The introduction of all thiols significantly decreased the stiffness of PEGDMA hydrogels with Young’s modulus values falling from 9.17 MPa for PEGDMA to 3.8, 3.9, 1.6 and 0.9 MPa for DiPETMP, PETMP, GDMP and ETTMP 1300, respectively. It should also be noted that both the GDMP and ETTMP 1300 have significantly lower stiffness values when compared to DiPETMP and PETMP, with the ETTMP 1300 samples also having significantly lower compressive strength measurements. In particular, ETTMP 1300 had a low mechanical strength, having approximately half the stiffness and a third of the compressive strength of the next weakest copolymer, GDMP. 

### 3.4. Wettability Measurements

**Figure 5:**
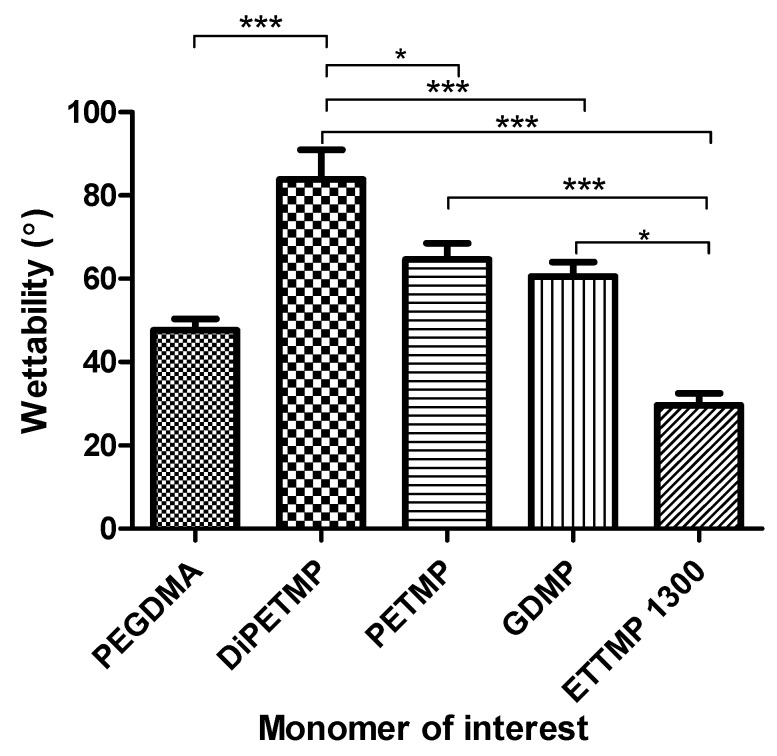
The wettability measurements of PEGDMA and PEGDMA-thiol samples.

From Figure 5, it can be seen that the introduction of thiols caused a general increase in hydrophobicity compared to pure PEGDMA. The PETMP and GDMP samples were found to have wettability measurements of 65° and 61° respectively, with the DiPETMP samples having a much more substantial increase in hydrophobicity, trending up to 84° and being significantly different to all other hydrogels. ETTMP 1300′s results were again in contrast to those of other thiols, increasing the hydrophilicity rather than hydrophobicity, with a wettability measurement of 30°. However, as the differences vary significantly between several of the thiol copolymers, this does open up the possibility of selecting different thiols to tune the wettability of a hydrogel toward a specific desired hydrophilicity. 

### 3.5. Thermal Properties

**Figure 6:**
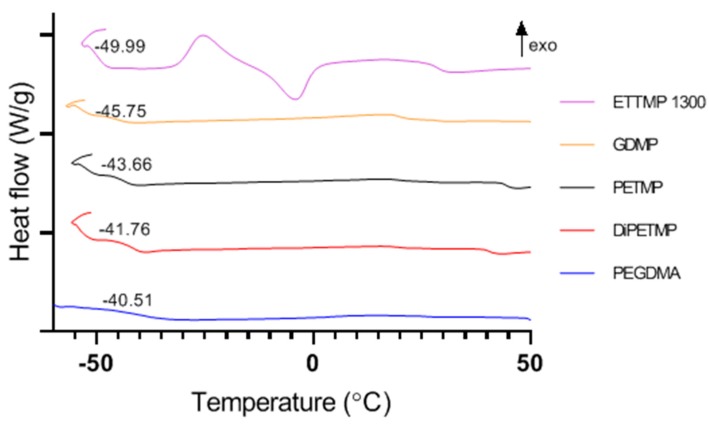
Differential scanning calorimetry highlighting the glass transition temperatures for PEGDMA and the different PEGDMA-thiol hydrogels.

A typical glass transition temperature (T_g_) for PEGDMA and PEGDMA-thiols can be seen in Table 3, with Figure 6 highlighting the typical DSC curves for each hydrogel. Of interest is that the hydrogels that seemed most likely to be fully polymerised based on previous results had the highest T_g_, namely PEGDMA, DiPETMP and PETMP. This is likely due to there being more fully reacted polymer chains, which increases the overall stiffness of the polymer network, necessitating more energy to increase the chain movement, resulting in a movement from a glassy to a rubbery state. This is supported by the Young’s modulus values in Section 3.3, where the stiffer the hydrogel is, the higher its eventual T_g_ value.

### 3.6. Surface Properties

**Figure 7:**
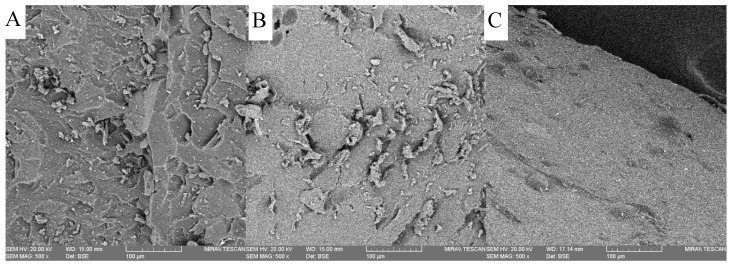

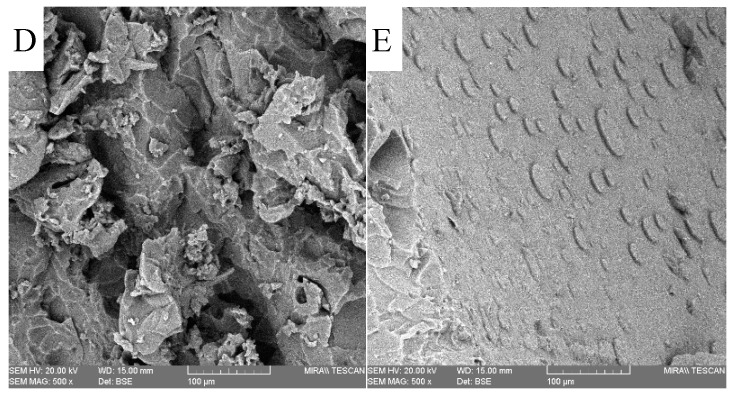
Scanning electron microscopy images of the surface topography of PEGDMA and PEGDMA-thiol hydrogels; (**A**) PEGDMA (**B**) DiPETMP, (**C**) PETMP, (**D**) GDMP and (**E**) ETTMP 1300.

The surface properties of the different thiol copolymers were examined using SEM analysis, and the results shown in Figure 7 below. When comparing the different thiol-ene hydrogels with pure PEGDMA, it appears that the surface morphology is not affected by the introduction of thiol (except in ETTMP 1300) as the hydrogel morphologies of PEGDMA, DiPETMP, PETMP and GDMP are very similar to one another. The morphology for ETTMP 1300 was markedly different when compared with the other hydrogels, with small circular protrusions covering the surface of the sample. These bubbles are most likely air bubbles formed during the mixing of ETTMP 1300 and PEGDMA, which were subsequently trapped in the hydrogel as it polymerised over time. ETTMP 1300′s differences, when compared with other hydrogels, as well as the fact that there is unreacted PEGDMA present throughout the polymer, led to the decision that further investigation of ETTMP 1300 copolymers would not be pertinent. An EDX analysis was carried out on all the samples, with carbon, oxygen and sulphur making up the vast majority of elemental responses across all samples.

### 3.7. Swelling and Gel Fraction Studies

**Figure 8:**
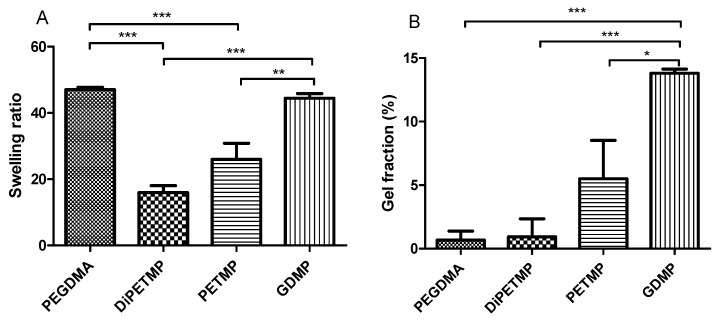
Comparison between (**A**) the swelling characteristics and (**B**) the gel fraction values of the PEGDMA and thiolated PEGDMA hydrogels.

The swelling ratio is an indicator of how tightly polymer networks are polymerised, with more considerable swelling showing a greater freedom between polymer chains. In Figure 8, DiPETMP and PETMP, which had similar stiffness values, have different swelling ratios; the DiPETMP samples, which have six functional groups compared to the PETMP samples’ four groups, are seen to have a swelling ratio of approximately 66% that of PETMP (swelling ratio of 15.93 compared with 25.97). As the thiol-ene ratio is done in a 1:1 reactive group ratio, this could be put down to the DiPETMP samples interacting with more PEGDMA linkages than the PETMP samples. This likely leads to faster rates of degradation, as there would be fewer PEGDMA-PEGDMA polymer chains linkages throughout the gel, which would be resistant to breakdown. GDMP copolymer networks have a swelling ratio comparable to PEGDMA (44.4 to 47.0), indicating that both samples have similar degrees of polymer network binding. With regard to pure PEGDMA, the large degree of swellability comes from every reacting monomer possessing a long kinetic chain, which gives the network freedom to expand. The GDMP copolymer, on the other hand, has a much shorter chain length and would therefore restrict the swelling of the hydrogel. When comparing the gel fraction results, it becomes clear that there was a large amount of unreacted monomer left in the GDMP hydrogel (13.8% gel) when compared to the PEGDMA hydrogel (0.7%); this more substantial degree of unreacted monomer could explain the comparability in swelling ratios between PEGDMA and GDMP. The relationship between GDMP’s gel fraction and swelling ratio is mirrored by the DiPETMP and PETMP samples, wherein a higher gel fraction corresponded to a higher swelling ratio. Of note is the comparability of the gel fraction values to the glass transition values, where the higher gel fraction values resulted in lower T_g_. Furthermore, the gel fraction values agreed with the previous results, reflecting which samples were most consistently polymerised, with PEGDMA being the most confidently polymerised and GDMP being the least confident.

### 3.8. Dynamic Mechanical Analysis

**Figure 9:**
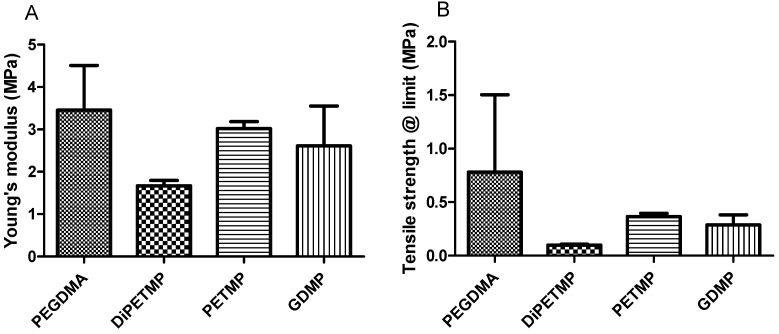
The dynamic mechanical analysis of the PEGDMA and PEGDMA-thiol samples post polymerization showing (**A**) Young’s Modulus and (**B**) the tensile strength at limit.

From the results in Figure 9, it could be seen that the inclusion of thiols reduced both the overall tensile strength at limit and tensile stiffness of the PEGDMA hydrogels in a similar manner to the effect that thiol inclusion had on the compressive strength at limit and stiffness. Unlike the compressive testing, where DiPETMP copolymers had values comparable to PETMP, in Figure 9 the largest decreases in tensile strength and stiffness occurred with the DiPETMP copolymer. With the PEGDMA, PETMP and GDMP samples, the results observed in Figure 9 matched more closely with the mechanical properties seen in Figure 4, namely with PEGDMA being the stiffest and strongest at limit, and PETMP having the most similar values. GDMP had values that were lower than both. These results differ in terms of the comparative differences between the samples in both tests. PEGDMA, which had almost three times the compressive strength and stiffness of PETMP (compressive strength of 9.17 to 3.94 MPa and stiffness of 4.05 to 1.09 MPa), dropped to less than twice the strength for the tensile measurements (tensile strength of 3.46 to 3.02 MPa and stiffness of 0.78 to 0.36 MPa). With regard to GDMP, the results are even more pronounced, with the tensile strength values actually increasing from compressive values of 1.6 MPa to a tensile strength at limit of 2.61 MPa, meaning that the GDMP samples go from having approximately one sixth the strength of the PEGDMA samples to having three quarters of PEGDMA’s strength.

**Figure 10:**
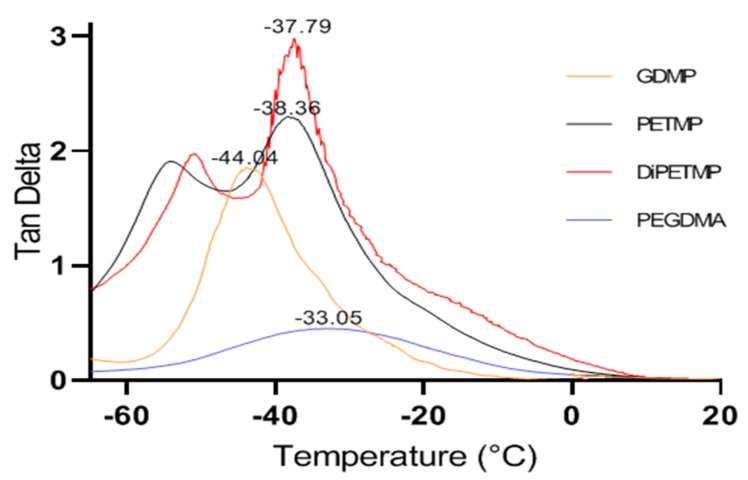
Dynamic mechanical analysis results showing tan delta for the PEGDMA and PEGDMA-thiol samples post polymerization.

From the tan δ peaks shown in Figure 10, all the thiol-PEGDMA composites had lower glass transition temperatures when compared to the PEGMDA samples, with the peaks shifting from –33.05 to –37.26, –38.29 or –44.04 °C for the DiPETMP, PETMP and GDMP copolymer networks, respectively. These results mirror the T_g_ values found with the DSC results from Section 3.5. One should note the appearance of a secondary tan δ peak for both the PETMP and DiPETMP samples, as well as what appeared to be secondary tan δ peaks for the PEGDMA and GDMP samples. These secondary peaks indicate that there are β relaxations occurring in these samples. This data matches what was observed in the DSC results in Figure 6, where there appeared to be β relaxations for the DiPETMP, PETMP and GDMP samples, as well as what could be a β relaxation for PEGDMA. The DMA and DSC results for PEGDMA are interesting, as the gap between the β relaxation and Tg is larger than for all the thiol samples in both tests, with PEGDMA occupying both the highest Tg values (representative of a polymers relaxation from a glassy to rubbery state) and also the lowest β relaxations (indicative of side chain motions in a polymer). Looking at the results above 0 °C, there do not appear to be any thermal events occurring in either pure PEGDMA or the thiol-based hydrogels.

### 3.9. Raman Spectroscopic Analysis

**Figure 11:**
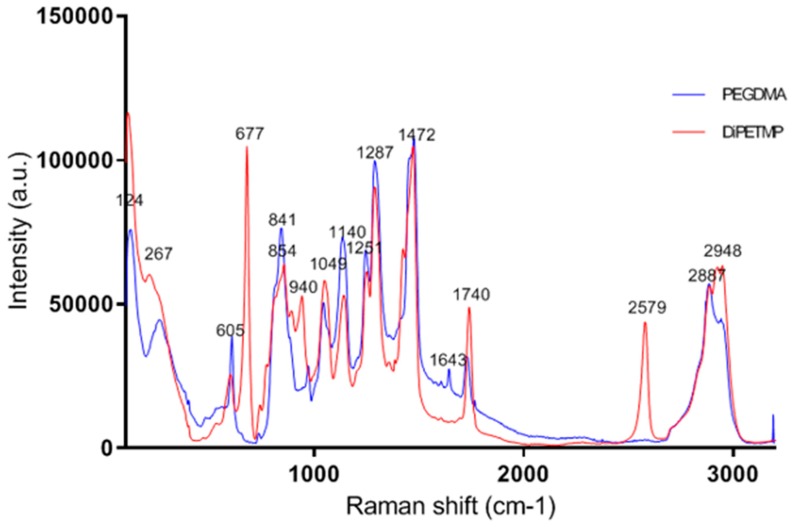
The Raman spectra for the PEGDMA and PEGDMA-DiPETMP samples.

From the previous testing, it was decided that the DiPETMP samples showed the most promise for future applications, due to their similarity of compressive strength with PEGDMA, degree of polymerisation and likelihood of an enhanced degradability in comparison to other thiol copolymer networks. With this in mind, a further chemical characterisation was carried out via the Raman spectroscopy of the PEGDMA and DiPETMP polymerised samples, as shown in Figure 11. As can be seen, the disappearance of the peak at 1640 cm^-1^ highlights the disappearance of all C=C bonds, indicating that the reaction between PEGDMA and DiPETMP resulted in a more complete crosslinking of the PEGDMA monomer than when PEGDMA was polymerized individually. Interestingly, in contrast to this, the peaks at 940 and 2580 cm^−1^ suggest that there are still thiol bonds present throughout the monomer [37], indicating that DiPETMP did not fully polymerize when reacting with PEGDMA. This may be due to the polymerization of the DiPETMP samples locking the partially reacted DiPETMP in the polymer network, preventing the continued polymerization with further PEGDMA samples; this is possible as DiPETMP selectively reacts with PEGDMA, as shown by the lack of peaks at 480–550 cm^−1^ associated with S– S bonding [38], whereas PEGDMA maintains the ability to react with either DiPETMP or other -ene functionalities.

### 3.10. Accelerated Degradation Study

**Figure 12:**
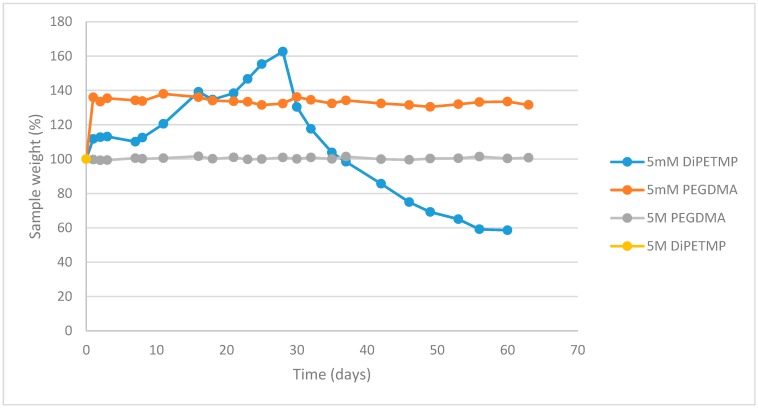
Degradation study of the PEGDMA and DiPETMP samples stored in 1. 5M NaOH and 2. 5 mM NaOH at 37 °C, over a 63 days period.

When carrying out an analysis of the weight change with 5M NaOH, the difference between PEGDMA and DiPETMP can be readily seen. The degradation of the samples over 70 days is shown in Figure 12. With the PEGDMA samples, there was no weight change from day 1 to the end of the experiment (day 63). The DiPETMP samples reached a complete breakdown, to the extent that all of the gel had been converted into a liquid within the first 24 hours of exposure. With regard to the 5 mM NaOH, the PEGDMA samples swelled to approximately 140% the weight of the day 0 samples and maintained that weight throughout the experiment. During the first month, the properties of DiPETMP in 5 mM NaOH did not change substantially, with the differences between PEGDMA and DiPETMP being those determined previously throughout this study. After a month, however, the mechanical properties of the hydrogels started to steadily decrease, as noted by increasing degrees of softness when removing the samples from the solution and blotting them dry. By day 63, the samples had reached a state of complete breakdown, with the retrieval from the solution invariably leading to damage to the hydrogel (see Table 4). Furthermore, attempts to blot dry DiPETMP samples led to the complete breakdown of the hydrogel structure. Therefore, the samples were unable to be weighed accurately for the day 63 testing, with one DIPETMP sample (S5) having reached a state of being irretrievable from the solution in order to be blotted dry. 5 mM NaOH allowed for a more gradual breakdown of hydrogel than 5M NaOH did, as the thiol hydrogels breakdown is directly proportional to the increase in present OH ions [11], i.e., as 5 mM NaOH has a concentration that is one-thousandth that of 5M NaOH, the rate of degradation should also be 1000 times slower. It is likely that as DiPETMPs swelling increased, numerous bonds on the DiPETMP sections of the monomer network were broken through hydrolytic degradation; as the PEGDMA portions were resistant to degradation and there were still sections of DiPETMP with an intact structure, the initial outcome was increased hydrogel swelling. Eventually, as more DiPETMP bonds were broken, the hydrogel would break down, resulting in a gradual decrease in weight until a complete breakdown was realised.

## 4. Discussion

From an initial group of ten thiols, only four thiols successfully polymerized with PEGDMA through either UV exposure or over time. All four thiols had mercaptopropionate functional groups. None of the monomers with mercaptoacetate functionalities reacted with PEGDMA to produce a hydrogel. It has been shown that mercaptoacetates are less reactive than mercaptopropionates, which may explain why there was no formation of thiol-ene copolymers [7]. However, it was also shown that the differences in the curing rates between any -ene and either mercaptoacetate or mercaptopropionate was not significant [7]. It is possible that the weight % of thiol per thiol monomer has an impact on the successful thiol-ene polymerization, since three of the thiols that successfully underwent copolymerization were all in the range of 24.1 to 26.8 weight %, but this is also unlikely since TMPMP has an –SH weight % of 24, which is only 0.1% off from the value of DiPETMP. With regard to the molecular weight, the weights of thiols which formed polymers with PEGDMA ranged from 238 up to 1300 Da, showing no sign that polymerization was affected by Mw within this range. The same appears to be true for functional group numbers which ranged from 2 to 6 in hydrogel producing thiols, although it is worth noting that the thiols with higher numbers of functional groups (DiPETMP with 6 and PETMP with 4) would present consistently higher compressive strengths than those of GDMP (2 functional groups) and ETTMP 1300 (3 functional groups). 

While all the stiffness values still fell in the range of values associated with mesenchymal stem cells differentiating into bone cells (ETTMP 1300 is on the border between bone and muscle cell differentiation) [16], there was a significant drop in stiffness following the introduction of all thiol monomers. Through adjustments to the ratios of the thiol-PEGDMA mixtures, it may be possible to lower the stiffness values to those necessary for muscle cell differentiation [39]. Another possible explanation for the difference in the mechanical strength of the PEGDMA-thiol copolymer networks, when compared to the PEGDMA hydrogels, could be the degree of polymer homogeneity. In pure PEGDMA samples, the polymer architecture consists of a homologous repetition of PEGDMA monomers that have a long kinetic chain length, limiting their ability to disperse stress. As the polymer architecture of thiol-ene copolymer networks is more heterogeneous, this lends itself to lower degrees of brittleness when compared to methacrylate hydrogels [40,41]. However, if this was true, ETTMP 1300 that has long kinetic chains should also have high degrees of stiffness and strength. This could be accounted for by the idea that ETTMP 1300 had an incomplete polymerisation leading to unreacted PEGDMA being present in both samples, as fully polymerised PEGDMA would have provided stiffer hydrogels.

The introduction of thiols to the PEGDMA polymer network had a marked impact on wettability. Wettability is an important factor in relation to tissue engineering, as samples that are too hydrophilic or too hydrophobic will inhibit cell attachment to their surface, with 40° to 60° being the ideal range for cellular adhesion [42]. As can be seen in Figure 5, the wettability values for pure PEGDMA hydrogels fell within the preferred wettability range, at 48°. As the wettability measurements of DiPETMP, PETMP and GDMP were found to be outside the ideal range for cellular adhesion, tissue engineering applications may be limited. To combat this, a number of options are possible and have been explored with regard to other polymer networks, including adding a more hydrophilic outer coating to the thiol-ene co-polymer or adding an additive to improve wettability [43,44]. In Table 3, the introduction of thiols can be seen to also heavily impact the glass transition temperature of PEGDMA, as all thiols lowered the needed temperature to allow for the necessary chain relaxations. This supports the idea that thiol-ene networks allow for softer hydrogels with a lower useful temperature range when compared to methacrylate networks due to the effect that thiol addition has on the overall heterogeneity of the polymer network [40]. The ETTMP 1300 samples’ results did not match those of other thiols, producing a Tm peak close to 0 °C (indicating the presence of water, which is supported by a large O–H stretch peak at 3400 cm^−1^ in the FTIR spectra of ETTMP 1300 [45]) in the sample, as well as a secondary T_g_ at 28 °C. This suggests that despite the 2 h mixing prior to attempted polymerisation there are miscibility issues between PEGDMA and ETTMP 1300, allowing the two monomers to separate while they were curing over time and were no longer being stirred.

The evidence supporting that ETTMP and PEGDMA have miscibility issues may be further supported by the SEM images, wherein the ETTMP 1300 samples appear to have bubbles appearing on their surface; these could be due to the previously mentioned presence of bubbles in the ETTMP 1300-PEGDMA mixtures or could indeed be a sign of the separation of ETTMP 1300 and PEGDMA. This, in addition to the observed shortcomings in the performance when compared to other thiol-ene polymers, as well as the failure to achieve complete polymerization, resulted in ETTMP-1300 being excluded from further investigation. The sole focus shifted to DiPETMP, PETMP and GDMP monomers and the study of the gel fraction. Figure 8B highlighted the DiPETMP samples as having the lowest degree of unreacted monomer, followed by PETMP. The GDMP thiol-ene networks were shown to have 13.8% gel, compared to PEGDMA’s 0.7%. Considering the previous FTIR results, it became clear that DiPETMP and PETMP were the most compatible hydrogels for a reaction with PEGDMA, with both thiols providing opportunities to impact the mechanical properties and wettability of PEGDMA samples while also improving their degradability.

When considering the mechanical properties, compared to other thiol-ene copolymers, the lower tensile strength of DiPETMP shown in Figure 9A is unexpected, as the DiPETMP copolymers appeared up to this point to match the PEGDMA hydrogel most closely. Furthermore, with a gel fraction percentage of 0.9%, the DiPETMP samples were second only to the PEGDMA polymer networks in confidence of polymerisation. As the tensile strength is related directly to crosslinking [46], DiPETMP was predicted to have an increased tensile strength when compared to hydrogels with lower degrees of crosslinking, such as GDMP. Furthermore, the SEM samples do not indicate any porosity within the sample, which could cause localised stresses and decrease the DiPETMP samples’ overall strength [47]. The DiPETMP mechanical performance and thermal properties matched more closely with PEGDMA than with either GDMP or PETMP. DIPETMP also provides a promising partner for copolymerisation with PEGDMA, with the aim to enhance degradability. The Raman results agree with what was seen with the gel fraction outcomes, namely that a high degree of polymerisation occurs in both PEGDMA-DiPETMP copolymerisation and in PEGDMA’s individual polymerisation; however, it also indicates that the PEGDMA-DiPETMP copolymer may have unreacted thiol groups remaining. As such, an accelerated degradation study of DiPETMP hydrogels was carried out. Through the addition of DiPETMP, the thiol copolymer networks achieved a complete breakdown following immersion in 5 mM NaOH for 9 weeks and 5 M NaOH for less than 24 h. This was compared with the fact that there was no change in the sample size or weight when the pure PEGDMA samples were placed in 5 M and 5 mM NaOH for the same period of time, highlighting DiPETMP’s ability to improve the degradability of PEGDMA-based hydrogels. Considering the differences in properties between PETMP and DiPETMP, DiPETMP can provide an option for improving the degradability of PEG-based hydrogels with different properties to PETMP.

## 5. Conclusions

From the initial testing of this study, it was determined that molecular weight had no discernible impact on whether a thiol monomer successfully polymerised with PEGDMA; additionally, it was shown that no mercaptoacetate-based monomer polymerised successfully with PEGDMA. When examining the material properties, PEGDMA-DiPETMP polymers were shown to have analogous or preferable properties when compared to PEGDMA-PETMP-based samples. When comparing the degradation rates of PEGDMA and DiPETMP-PEGDMA polymers, it could be seen that the introduction of DiPETMP greatly increased PEGDMA’s degradability.

## Figures and Tables

**Table 1:** Combination of monomers in 1:1 molar ratios.

Monomer	PEGDMA (g)	Thiol (g)	Photoinitiator (mg)
PEGDMA	13.00	0.00	13.00
PETMP	7.00	5.70	12.70
DiPETMP	5.20	7.88	13.08
PETMA	5.00	8.60	13.60
ETTMP 700	4.00	9.33	13.33
ETTMP 1300	2.50	10.83	13.33
TMPMP	5.50	7.31	12.81
TEMPIC	5.00	8.76	13.76
GDMA	10.00	3.50	13.50
GDMP	9.00	3.77	12.77
PCL4MP	4.00	9.00	13.00

**Table 2:** Results of the PEGDMA-thiol polymerisation check.

Monomer	Reactive Groups	Molecular Weight	SH Content (%) [30]	Polymerization Success (Yes/No)
PETMP	4	489	26.0	Yes
DiPETMP	6	909	24.1	Yes
PETMA	4	433	29.5	No
ETTMP 700	3	700	13.5	No
ETTMP 1300	3	1300	7.1	Yes
TMPMP	3	399	24.0	No
TEMPIC	3	526	18.4	No
GDMA	2	210	30.5	No
GDMP	2	238	26.8	Yes
PCL4MP	4	1350	9.1	No

**Table 3:** The glass transition temperatures of thiolated PEGDMA hydrogels (n = 2).

Monomer	Mean T_g_ (°C)
PEGDMA	−39.04 ± 1.82
DiPETMP	−41.88 ± 0.95
PETMP	−43.47 ± 0.27
GDMP	−45.66 ± 0.13
ETTMP 1300	−49.86 ± 0.18

**Table 4:** The changes to the hydrogel morphology during the accelerated degradation study, from weeks 4 to 8.

Monomer of Interest (NaOH Concentration)	Week 4	Week 5	Week 6	Week 7	Week 8
PEGDMA (5 M)	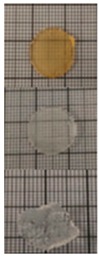	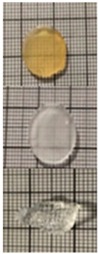	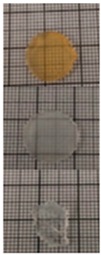	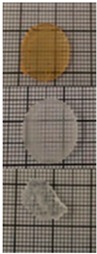	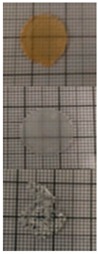
PEGDMA (5 mM)					
DiPETMP (5 mM)					

## References

[1] Murugan R., Ramakrishna S. (2006). Nano-Featured Scaffolds for Tissue Engineering: A Review of Spinning Methodologies. Tissue Eng..

[2] Fourniols T., Randolph L.D., Staub A., Vanvarenberg K., Leprince J.G., Préat V., des Rieux A., Danhier F. (2015). Temozolomide-loaded photopolymerizable PEG-DMA-based hydrogel for the treatment of glioblastoma. J. Control. Release.

[3] El-Sherbiny I.M., Yacoub M.H. (2015). Hydrogel scaffolds for tissue engineering: Progress and challenges Ibrahim. Glob. Cardiol. Sci. Pract..

[4] Ifkovits J.L., Burdick J.A. (2007). Review: Photopolymerizable and Degradable Biomaterials for Tissue Engineering Applications. Tissue Eng..

[5] Patterson J., Hubbell J.A. (2010). Enhanced proteolytic degradation of molecularly engineered PEG hydrogels in response to MMP-1 and MMP-2. Biomaterials.

[6] Nair L.S., Laurencin C.T. (2007). Biodegradable polymers as biomaterials. Prog. Polym. Sci..

[7] Morgan C.R., Magnotta F., Ketley A.D. (1977). Thiol/Ene Photocurable Polymers. J. Polym. Sci. Polym. Chem. Ed..

[8] Cramer N.B., Reddy S.K., O’Brien A.K., Bowman C.N. (2003). Thiol-Ene Photopolymerization Mechanism and Rate Limiting Step Changes for Various Vinyl Functional Group Chemistries. Macromolecules.

[9] Zhou J., Zhang Q.Y., Chen S.J., Zhang H.P., Ma A.J., Ma M.L., Liu Q., Tan J.J. (2013). Influence of thiol and ene functionalities on thiol-ene networks: Photopolymerization, physical, mechanical, and optical properties. Polym. Test..

[10] Lu H., Stansbury J.W., Bowman C.N. (2005). Impact of Curing Protocol on Conversion and Shrinkage Stress. J. Dent. Res..

[11] Shih H., Lin C. (2012). Crosslinking and degradation of step-growth hydrogels formed by thiol-ene photo-click chemistry. Biomacromolecules.

[12] Rydholm A.E., Bowman C.N., Anseth K.S. (2005). Degradable thiol-acrylate photopolymers: Polymerization and degradation behavior of an in situ forming biomaterial. Biomaterials.

[13] Lowe A.B. (2014). Thiol-ene ’click’/coupling chemistry and recent applications in polymer and materials synthesis and modification. Polymer (Guildf).

[14] Manzo M., Ioppolo T. (2015). Untethered photonic sensor for wall pressure measurement. Opt. Lett..

[15] Aimetti A.A., Machen A.J., Anseth K.S. (2009). Poly(ethylene glycol) hydrogels formed by thiol-ene photopolymerization for enzyme-responsive protein delivery. Biomaterials.

[16] Das R.K., Zouani O.F. (2014). A review of the effects of the cell environment physicochemical nanoarchitecture on stem cell commitment. Biomaterials.

[17] Guilak F., Cohen D.M., Estes B.T., Gimble J.M., Liedtke W., Chen C.S. (2009). Control of Stem Cell Fate by Physical Interactions with the Extracellular Matrix. Cell Stem Cell.

[18] Reilly G.C., Engler A.J. (2010). Intrinsic extracellular matrix properties regulate stem cell differentiation. J. Biomech..

[19] Cramer N.B., Davies T., O’Brien A.K., Bowman C.N. (2003). Mechanism and modeling of a thiol-ene photopolymerization. Macromolecules.

[20] Burget D., Mallein C., Fouassier J.P. (2004). Photopolymerization of thiol-allyl ether and thiol-acrylate coatings with visible light photosensitive systems. Polymer (Guildf).

[21] Oesterreicher A., Wiener J., Roth M., Moser A., Gmeiner R., Edler M., Pinter G., Griesser T. (2016). Tough and degradable photopolymers derived from alkyne monomers for 3D printing of biomedical materials. Polym. Chem..

[22] Podgórski M., Becka E., Chatani S., Claudino M., Bowman C.N. (2015). Ester-free thiol-X resins: New materials with enhanced mechanical behavior and solvent resistance. Polym. Chem..

[23] Trey S.M., Nilsson C., Malmström E., Johansson M. (2010). Thiol-ene networks and reactive surfaces via photoinduced polymerization of allyl ether functional hyperbranched polymers. Prog. Org. Coat..

[24] Burke G., Barron V., Geever T., Geever L., Devine D.M., Higginbotham C.L. (2019). Evaluation of the materials properties, stability and cell response of a range of PEGDMA hydrogels for tissue engineering applications. J. Mech. Behav. Biomed. Mater..

[25] Killion J.A., Kehoe S., Geever L.M., Devine D.M., Sheehan E., Boyd D., Higginbotham C.L. (2013). Hydrogel/bioactive glass composites for bone regeneration applications: Synthesis and characterisation. Mater. Sci. Eng. C.

[26] Hurley D., Carter D., Lawrence N., Davis M., Walker G.M., Lyons J.G., Higginbotham C.L. (2019). An investigation of the inter-molecular interaction, solid-state properties and dissolution properties of mixed copovidone hot-melt extruded solid dispersions. J. Drug Deliv. Sci. Technol..

[27] Bäckström S., Benavente J., Berg R.W., Stibius K., Larsen M.S., Bohr H., Helix-Nielsen C. (2012). Tailoring Properties of Biocompatible PEG-DMA Hydrogels with UV Light. Mater. Sci. Appl..

[28] Browning M., Cereceres S., Luong P.T., Cosgriff-Hernandez E. (2014). Determination of the in vivo degradation mechanism of PEGDA hydrogels. J. Biomed. Mater. Res. Part A.

[29] Lam C.X.F., Savalani M.M., Teoh S.H., Hutmacher D.W. (2008). Dynamics of in vitro polymer degradation of polycaprolactone-based scaffolds: Accelerated versus simulated physiological conditions. Biomed. Mater..

[30] Bruno Bock Chemische Fabrik GmbH & Co, KG (2012). BrunoBock Product Catalogue.

[31] Wu Y.H., Park H.B., Kai T., Freeman B.D., Kalika D.S. (2010). Water uptake, transport and structure characterization in poly(ethylene glycol) diacrylate hydrogels. J. Membr. Sci..

[32] Decker C., Moussa K. (1987). Photopolymerization of multifunctional monomers in condensed phase. J. Appl. Polym. Sci..

[33] Dobić S.N., Filipović J.M., Tomić S.L. (2012). Synthesis and characterization of poly(2-hydroxyethyl methacrylate/itaconic acid/poly(ethylene glycol) dimethacrylate) hydrogels. Chem. Eng. J..

[34] Silverstein R.M. (1998). Webster Spectrometric Identification of Organic Compounds.

[35] Coates J. (2000). Interpretation of Infrared Spectr A Practical Approach Interpretation of Infrared Spectra A Practical Approach. Encycl. Anal. Chem..

[36] Miller F.A., Wlkins C.H. (1952). Infrared Spectra and Characteristic Frequencies of Inorganic Ions. Anal. Chem..

[37] Bazylewski P., Divigalpitiya R., Fanchini G. (2017). In situ Raman spectroscopy distinguishes between reversible and irreversible thiol modifications in l-cysteine. RSC Adv..

[38] Schulz H., Baranska M. (2007). Identification and quantification of valuable plant substances by IR and Raman spectroscopy. Vib. Spectrosc..

[39] Even-Ram S., Artym V., Yamada K.M. (2006). Matrix Control of Stem Cell Fate. Cell.

[40] Ligon S.C., Liska R., Stampfl J., Gurr M., Mülhaupt R. (2017). Polymers for 3D Printing and Customized Additive Manufacturing. Chem. Rev..

[41] Ligon-Auer S.C., Schwentenwein M., Gorsche C., Stampfl J., Liska R. (2016). Toughening of photo-curable polymer networks: A review. Polym. Chem..

[42] Arima Y., Iwata H. (2007). Effect of wettability and surface functional groups on protein adsorption and cell adhesion using well-defined mixed self-assembled monolayers. Biomaterials.

[43] Zhong S.-P. (1997). Method of Providing A Substrate With Hydrophilic Coating and Substrates, Particularly Medical Devices, Provided With Such Coatings. U.S. Patent.

[44] Otitoju T.A., Ahmad A.L., Ooi B.S. (2018). Recent advances in hydrophilic modification and performance of polyethersulfone (PES) membrane via additive blending. RSC Adv..

[45] Moffat A.C., Osselton D.M., Widdop B., Watts J. (2011). Clarke’s Analysis of Drugs and Poison.

[46] Pedro R.N., Hendlin K., Kriedberg C., Monga M. (2007). Wire-Based Ureteral Stents: Impact on Tensile Strength and Compression. Urology.

[47] Liu X., Won Y., Ma P.X. (2006). Porogen-induced surface modification of nano-fibrous poly(l-lactic acid) scaffolds for tissue engineering. Biomaterials.

